# The Cause of Milk-Sickness

**Published:** 1861

**Authors:** S. C. Chase

**Affiliations:** Harmer, Ohio


### THE CAUSE OF MILK-SICKNESS.

BY S. C. CHASE, M.D., Harmar, Ohio.

MESSRS. EDITORS:—I find in the March number of your journal an address on the above subject, by Isaac Mendenhall, M. D., of Ashland, Ind. Nor was I aware until I saw his essay, that the cause of this malady was unknown, generally, to the medical faculty. I have been a practitioner fourteen years, during all of which time I have regared the cause of milk-sickness as easily traceable to its origin as the cause of ague, or any of the endemics; with this difference, that while the cause of endemics usually depend upon atmospheric conditions, the presence of miasmata, etc., the cause of milk-sickness depends upon the presence of vegetation, which abounds in certain localities only; for which reason the cattle of the fallow ground pasture field are exempt, while those of the adjacent inclosed wood pasture are trembling with the disease. In such wood pasture will be found the rhus toxicodendron growing every where among the grass, so that if the cattle eat grass they must also eat rhus. Notwithstanding instinct enables dumb animals to avoid some poisonous plants, yet in dry seasons cattle will devour the rhus with avidity, and have no objections to the foliage of the buckeye, the latter of which often produces as serious consequences (as far as the animal is concerned] as the former.

In the year 1841-2 I resided near the little village of Wyandot, in Marion County, O., during which time there were several cases of milk sickness in the neighborhood, some of which were fatal. The people, of course, were alarmed; milk, butter, and fresh meats, beef and veal were considered dangerous. As the excitement increased, the question with every body was, what is the cause? To solve the mystery, Dr. Brown, of Wyandot, and myself, commenced an investigation. In this undertaking I consider we were favorably located, as the surroundings were such as to afford us an ample opportunity to trace the effect to the cause, as there were but two places for the cattle to drink, and but three kinds of pasture; the first of which—viz: the inclosed, cultivated field, was exempt, while the contiguous inclosed, uncultivated wood pasture, and the prairie pasture contained the cause, as the cattle drank from troughs, supplied from wells, or at the Sandusky river, which is near the village. The water could not be the cause, as the cattle in the cultivated fields drank of the same water in both cases. As the cause was not atmospheric, we were forced to the conclusion that it was something which the cattle ate. After examining the whole premises carefully, and finding no other poisonous vegetable, we were satisfied that rhus was the cause of the disease,—in the texicology of which we believe the entire phenomena, as well as the pathological condition, (as present in milk-sickness,) will be found.

On breaking a stem of the rhus-leaf a milk-like fluid is exuded, which is exceedingly poisonous; if applied to the skin it will produce effects similar to those of argentum nitras, a black welt is produced, in a few hours becoming sore, destroys the cuticle, which sloughs, and upon healing leaves a circular cicatrice. So poisonous is rhus that it pollutes the atmosphere where it grows, so that children, as well as grown persons, who go among it to gather berries from bushes growing in the same locality, are badly poisoned. I have seen their faces swelled until their eyes were shut, their necks, hands and arms covered with inflamed vesicles, the cuticle highly inflamed, and not unfrequently constitutional symptoms produced, resembling those of milk-sickness. The nostrils of cattle that graze among it are covered with pustules. In fact, there can be no doubt that its poisonous exhalations are more to be feared than those of the fabled upas. Who can doubt that such quantities as are consumed by cattle must poison their blood, contaminate the milk, and be present in the butter, and produce all the symptoms enumerated by Dr. Mendenhall? That the milk and butter of such animals should be poisonous is in unison with the relation of cause and effect. And now, in summing up the evidence, we would respectfully present the following facts in the premises: First, there was no rhus in the cultivated field, (as cultivation destroys it,) and the cattle were not affected. Secondly, the contiguous wood pasture was covered with rhus, the cattle ate it, and were diseased. Thirdly, there was no other poisonous plant in either pasture. Fourthly, there was no water in either pasture, and the cattle inhaled the same air, except the atmosphere as impregnated by rhus. Will some of the physicians who reside where there is milk-sickness, please test the influence of rhus toxicodendrun, as the suspected cause, by penning up a few cattle and feeding them for a few days on rhus only, and report through your journal?—*Cin. Lancet and Observer.*

